# Preparation, optimization, and characterization of genistein-ginseng long-acting polymeric gel as a breast cancer treatment alternative

**DOI:** 10.1007/s12672-024-01132-8

**Published:** 2024-07-03

**Authors:** Samaa Abdullah, Shadab Md, Abeer A. Altamimi, Hadil Alahdal, Raisuddin Ali, Huda Mohammed Alkreathy, Shahid Karim

**Affiliations:** 1 Natural and Health Sciences Research Centre, Princess Nourah bint Abdulrahman University, P.O. Box 84428, 11671 Riyadh, Saudi Arabia; 2 Department of Pharmaceutics, Faculty of Pharmacy, King Abdulaziz University, Jeddah, Saudi Arabia; 3 Department of Biology, Faculty of Science, Princess Nourah Bint Abdulrahman University, 84428 Riyadh, Saudi Arabia; 4 Department of Pharmaceutics, College of Pharmacy, King Saud University, Riyadh, Saudi Arabia; 5 Department of Clinical Pharmacology, Faculty of Medicine, King Abdulaziz University, Jeddah, Saudi Arabia

**Keywords:** Genistein, Ginseng, Solid dispersion, In-situ gelling, Penetration, Dissolution enhancement

## Abstract

To address the prevalent genistein (GST) metabolism and inadequate intestinal absorption, an oral long-acting and gastric in-situ gelling gel was designed to encapsulate and localize the intestinal release of the loaded genistein-ginseng (GST-GNS) solid dispersion. Because of the high breast perfusion of GST upon oral absorption, the GST-GNS solid dispersion was developed to enhance GST's dissolution and penetration while offering a synergistic impact against breast cancer (BC). Physiochemical analysis of the GST-GNS solid dispersion, release analysis, gel characterizations, storage stability, penetration, and in vitro cytotoxicity studies were carried out. GST-GNS solid dispersion showed improved dissolution and penetration as compared to raw GST. GST-GNS solid dispersion homogenous shape particles and hydrophilic contacts were revealed by scanning electron microscopy and Fourier Transform-Infrared analysis, respectively. GST-GNS solid dispersion’s diffractogram shows the amorphous character. A second modification involved creating a gastric in-situ gelling system loaded with GST-GNS solid dispersion. This system demonstrated improved GST penetration employing the solid dispersion, as well as the localizing of the GST release at the intestinal media and antitumor synergism against BC. For a better therapeutic approach for BC, the innovative oral GST long-acting gel encasing the GST-GNS solid dispersion would be recommended.

**Graphical Abstract:**

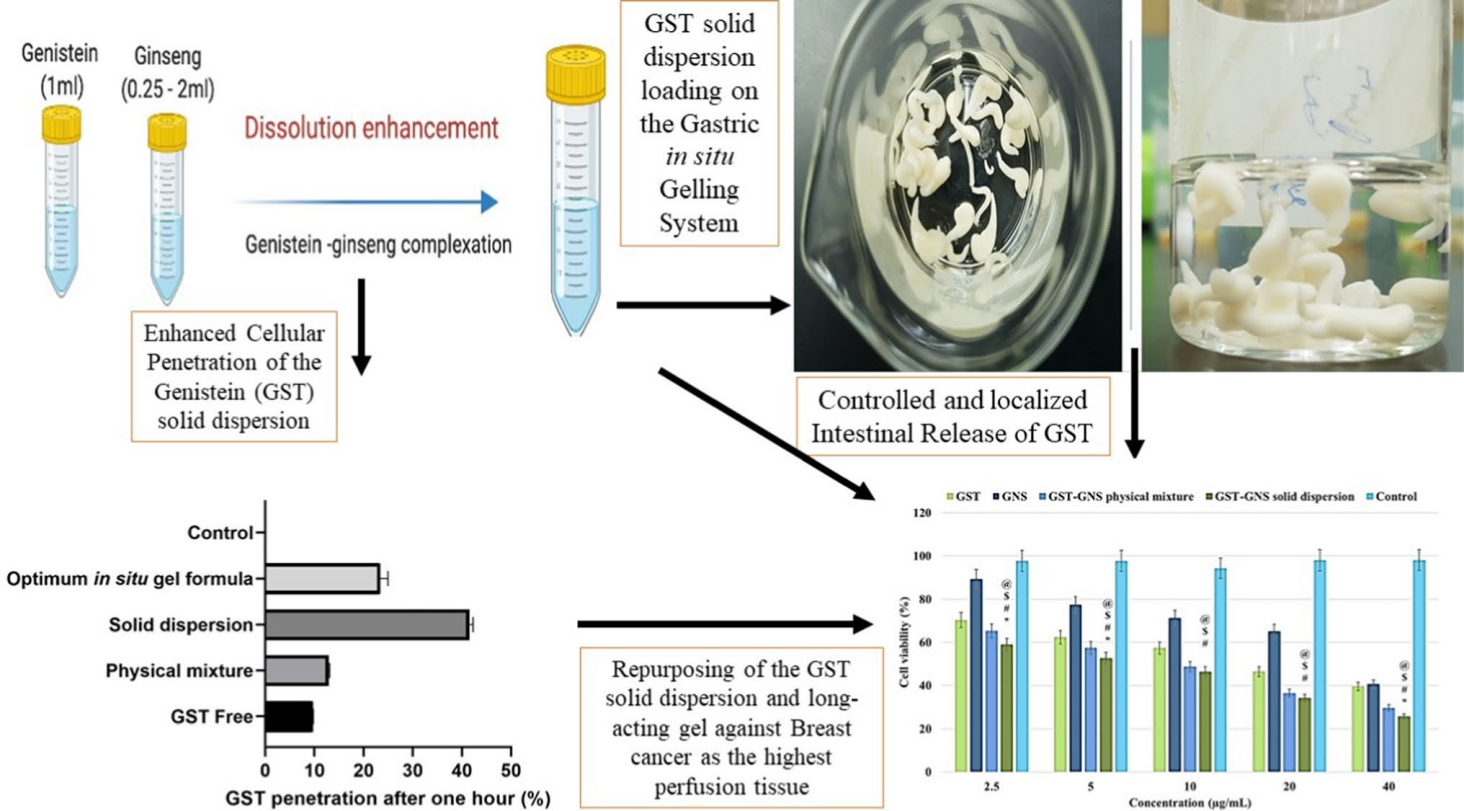

**Supplementary Information:**

The online version contains supplementary material available at 10.1007/s12672-024-01132-8.

## Introduction

Breast cancer (BC) is among women's most commonly diagnosed cancer types, apart from non-melanoma [1]. As per the published reports, more than 2.3 million women were diagnosed with BC, and approximately 685,000 deaths were reported worldwide. Additionally, the epidemiological data showed that by the end of 2020, approximately 7.8 million women with confirmed cases of BC existed. This accounts for BC as the most prevalent type of cancer [1]. BC is diagnosed in all grouped women but primarily women above the age of 45 [2]. More than half of cases of BC develop without any predictable risk factors [3].

However, despite the profound use of adriamycin, docetaxel, methotrexate, 5-fluorouracil, cyclophosphamide, ribociclib, abemaciclib, trastuzumab, and palbociclib, most patients have a poor quality of life and clinical outcome. Additionally, these drugs exhibit severe side effects and suffer from pharmacokinetic limitations. Hence, there is an unmet need for an alternative therapeutic approach to overcome these limitations and exhibit a potent anticancer effect [4].

Genistein (GST) is one of the extensively studied isoflavones and is reported to exhibit potent antioxidant, anti-inflammatory and anticancer properties. Various studies reported anticancer activity against BC by different molecular mechanisms, such as causing apoptosis evasion by reducing mRNA with modulation of E2F1, arresting cell cycle in G_0_/G_1_ phase, and antiproliferation through minimizing DNA methylation [5, 6]. Despite its multiple pharmacological attributes, GST presents the limitation of low solubility, poor absorption and fast hepatic metabolism [5, 6]. Against cancer, Ginseng (GNS) exhibited its mechanism via various pathways, such as antiproliferation through inhibition of oncogenes c-mycs, c-fos and nucleophosmin downregulation, apoptosis (inhibition of TNF-α, and ERK pathways, modulation of mitochondrial cyt C, and poly ADP ribose polymerase), and cell cycle regulation through inhibition of MMP-2 and 9 [7, 8]. However, GNS’s ginsenosides are highly metabolized by the first-pass effect [9]. According to the reports, GST and GNS were studied individually and scientifically proven to be safe against normal cells [10, 11]. In addition, GST and GNS were studied individually for skin and wound healing, respectively [10, 12]. From these points, nutraceuticals could be considered safe anticancer candidates in comparison to the usual approved chemotherapeutics.

Thus, the GST-GNS solid dispersion was developed through solid dispersion to explore the synergistic anticancer and penetration effects. When poor soluble GST is coated with GNS using the GST-GNS solid dispersion, it could enhance the dissolution and penetration of GST via ginsenosides present in GNS [13]. Due to the extensive GST metabolism and poor intestinal absorption, an oral long-acting and gastric in situ gelling gel were optimized to encapsulate and localize the intestinal GST release of the GST-GNS solid dispersion, which the solid dispersion could show an enhanced dissolution and penetration compared to the raw GST particles [14]. Furthermore, the GNS coating of GST could lower the hepatic metabolism of GST after oral absorption due to the saturation of Cytochrome P450 by the GNS extract components [9, 15].

Therefore, in the present study, the GST-GNS solid dispersion was optimized and characterized using Fourier Transform-Infrared spectroscopy (FT-IR), Powder X-ray Diffractometer (PXRD), Scanning Electron Microscopy (SEM), GST content assay, GST solubility studies, and the GST release study in comparison to the GST, GNS, and physical mixture for them. In addition, the GST-GNS solid dispersion was studied to investigate the GST dissolution enhancement and penetration effects compared to the raw GST and physical mixture. Furthermore, the GST-GNS solid dispersion encapsulation in the optimized oral long-acting gel was based on the gastric in situ (insoluble) gelling system to localize the GST-GNS solid dispersion release at the intestinal region as the GST absorption site, in which the GST-GNS solid dispersion enhanced the GST’s dissolution and penetration.

## Materials and methods

### GST-GNS solid dispersion

A 0.10 mg/mL solution of GST (CAS.NO. 446-72-0, Merk, USA) and GNS (CAS.NO. 05115001, Merk, USA) was prepared in methanol. Then, each 0.25, 0.5, 0.75, 1, 1.5, and 2 mL of the GNS solution was collected in different vials and combined with 1 mL of the GST solution with continuous stirring (400 rpm for 5 min) using a magnetic stirrer. The opaque and physical alterations were noticed while mixing the different blended solutions [16–20].

### Solid dispersion isolation and GST content assay

The 1:1 ratio of GST: GNS solution was the only ratio to provide a long-lasting opaque appearance after 5 min. The prepared solid dispersion was isolated through solvent evaporation using a rotary evaporator (REV100-P, Bioevopeak, China) at 75.00 ± 0.05 rpm and 40 ± 0.05  C [16]. Additionally, the obtained dry sample was milled using a mortar and pestle to collect the micronized particle of size 250 µm, using sieve filtration of 250 µm cut-off value. Furthermore, the GST content assay required mixing and vortexing to dissolve 5 mg of the solid dispersion in 50 mL methanol. After absolute solubilization with methanol, the GST concentrations in the clear solution were analyzed using a UV-spectrophotometer at 260 nm [21, 22].

### GST-GNS solid dispersion solubility assessment

In comparison to the GST raw material, the solubility of GST in 0.1 N HCl (pH 1.2) and phosphate buffer (pH 6.8) was measured for the optimum solid dispersion and its physical combination. Samples containing more than 2 mL of the fluid under investigation were kept in 15 mL glass tubes. Before being immersed in a shaking water bath for 72 h, the materials were vortexed for 15 min. The supernatant was completely dissolved in a particular amount of methanol after centrifuging the sample. A UV-spectrophotometer was used to measure the GST content at 260 nm [18–20, 22].

### The formulation and preparation of the long-acting gel encapsulating the GST-GNS solid dispersion

The optimum GST-GNS solid dispersion was mixed with different volumes of carrageenan-Lambda (CRG), 209 NF (Viscarin, USA), and 18 mg/mL of alginate (NA)-high molecular weight (600,000 g/mole, Sigma, USA) as mentioned in Table 1 using stirrer machine for 10 min [23].

**Table 1:** Gastric insoluble gel formulations of GST-GNS solid dispersion prepared at a GST concentration of 60 mg/ml.

#	NA^a^-gel (mg/ml)	CRG^b^-gel (mg/ml)	GNS (mg/mL)	GST (mg/mL)
F1	18 [23]	2	60	60
F2		4		
F3		8		

The total formulation volume was 10 mL

^a^Sodium alginate High molecular weight of 600,000 g/mole

^b^Carrageenan Lambda gel

### GST release analysis and optimization of the long-acting gel encapsulating the GST-GNS solid dispersion

The GST release was analyzed at a controlled temperature of 37.00 ± 0.05 °C in a water bath shaker with 75.00 ± 0.05 rpm in various samples, such as GST raw material, GST­GNS solid dispersion, GST-GNS dry physical mixture, and the different formulations in Table 1. The GST raw material, GST­GNS solid dispersion, GST-GNS dry physical mixture test groups, and the other formulations used amounts were equivalent to 600 mg in 10 mL total volume (GST concentration is 60 mg/mL) [19, 20, 22]. The formulation's entrapment efficiency values were based on the drug content assay of the GST-GNS solid dispersion (0.43 mg GST/1 mg solid dispersion). 1764.71 mg of the optimum solid dispersion was incorporated in the different in situ gelling formulations to incorporate 600 mg of GST in 10 mL total volume. The dispersion samples were placed in dialysis bags with a cut-off value of 12,000–14,000 (Sigma, USA) and immersed in 300 mL of the release media. For the first 2 h, the groups were immersed in 0.1 N HCl, a simulated stomach fluid with a pH of 1.2. The groups were then immersed in a phosphate buffer with a pH of 6.8, which resembled proximal intestinal fluid. To imitate the sink body conditions, the medium was completely replaced every hour. The samples were placed in an equal volume of methanol to avoid GST precipitation before measurement [23].

### Storage release stability testing of the optimum long-acting gel encapsulating the GST-GNS solid dispersion

The most optimum long-acting gel encapsulating the GST-GNS solid dispersion found in the previous section was kept in an amber glass container at room temperature (25.00 ± 0.05 °C) for 2 weeks and 4 weeks of storage to be compared with the zero-time prepared gel [29, 30]. At the time of analysis, the optimum formula was placed in the dialysis bag for the GST release analysis as mentioned in Sect. 2.5 [28].

### GST-GNS solid dispersion characterizations

#### Fourier transform-infrared (FT-IR)

FT-IR spectrum determination was carried out by scanning the 5–10 mg samples of GST, GNS, GST-GNS solid dispersion, and GST-GNS physical mixture between 500 and 4000 cm^−1^ (Bruker, Hyperion II, USA).

#### Powder X-ray diffractometer (PXRD)

The GST, GNS, GST-GNS solid dispersion and GST-GNS physical mixture were studied using a Maxima XRD-7000X PXRD system (Shimadzu, Kyoto, Japan). X-rays were produced at 40 kV and 100 mA during the procedure utilizing nickel-filtered Cu Kb reduction. The scan range (2θ) was 5 to 70 , with a speed of 10  per minute.

#### Morphology and dispersion investigations

Scanning electron microscopy (SEM, Zeiss, Germany) was used to examine the structure and particle distribution of GST, GNS, GST-GNS solid dispersion, and GST-GNS physical combination in powder form. The dried samples were evaluated using a 20 kV voltage.

### In situ gel characterizations

Measurements were made of the in situ/insoluble gels in Table 1's strength, volume, and resilience. The optimal gel for encasing the GST solid dispersion was selected using the gel characterizations and GST release. They assessed the gels' dimensions, weight, strength, and resilience after they were produced in 0.1 N HCl [23].

#### Gel volume and weight

150 ml of 0.1N HCl and a volume of 15 ml were put into a preweighed 250 ml glass beaker (W1). After the insoluble gel was produced for thirty minutes, the location of the top of the gel was marked on the outside of the beaker. Following the creation of an insoluble gel, the weight of the beaker and its contents (W2) was determined. After carefully decanting the supernatant liquid and transferring the insoluble gel into a pre-tared watch glass, the insoluble gel was gently removed from the beaker. After 30 s, the extra liquid was drained, and the insoluble gel was weighed (W3). Before the beaker was filled with water to the proper level and weighed, the remaining liquid was scraped from its interior using a paper towel (W4). With a supernatant density of 1, the volume (mL) of each insoluble gel was determined using the weights (gram) obtained as (W4–W1) − (W2–W1–W3) [24].

#### Insoluble gel resilience

Each formula (15 ml) was placed into 150 ml of 0.1 N HCl in a 50 mL centrifuge tube that was kept at 37 °C. To simulate stomach agitation, the tube was sealed after 30 min and rotated at 20 rpm in a roller mixer (Roller Mixer-205 RM, Hawashin Tech. Company, Korea). Visual evaluations of the insoluble gel were conducted at 2, 5, 10, 20, 30, 45, and a maximum of 120 min, or until it ceased to be visible. Insoluble gel resilience was calculated using the time at which the insoluble gel could no longer be seen [25].

#### Insoluble gel strength

For 30 min, insoluble gels were created in a 250 ml glass beaker by adding 15 mL of formulation volume to 150 mL of 0.1 N HCl that was kept at 37 °C. After the insoluble gels were taken out, a Texture Analyzer XT Plus C apparatus (Stable Micro Systems, UK) was used to measure the force needed for a stainless-steel cone to break the insoluble gel. Once the force necessary was divided by the stainless-steel cone's constant acceleration, the strength was represented in terms of mass (g) [25].

### The cell viability assay of the solid dispersion

The duly cultured cell lines were grown under 37 °C, 80–90% confluence, and 5% CO_2._ For the cell viability, the MTT assay kit of thiazolyl blue tetrazolium bromide reagent was procured from ABCAM, UK. For performing the assay of cell viability for the GST’s high perfusion tissue, BC cell line (MCF-7) and MTT proliferation kit were used. Cells were initially left for incubation at 37 °C for 24 h using a 96-well plate. In each well, 5 × 10^3 MCF-7 cells were placed and kept inside the humified carbon dioxide incubator, maintained at 5% humidity. The treatment groups such as GNS and GST, their physical mixture, and solid dispersion were added to the well at different concentrations and left for 24 h. After this, all the samples were centrifuged, and precisely 100 µl of obtained supernatant was replaced with that of DMSO and again left for incubation at 5% carbon dioxide and 37 °C for 4 h. The absorbance was recorded at 570 nm in a triplicate using a microplate reader [26].

### The mucus-penetrating study

The penetration ability of GST particles was assessed using an artificial mucus model that comprises a mucus layer placed over a gelatin layer [23, 27]. A 10% (w/v) gelatin solution was prepared in hot water to be placed in a 24-well plate at a volume of 1 mL per well. It was left to harden at room temperature. To prepare the mucus dispersion at room temperature, mix 250 mg mucin, 250 µL sterile egg yolk emulsion, 0.295 mg diethylene-tri-aminpenta-acetic acid (DTPA), 250 mg NaCl, and 110 mg KCl in 50 mL of distilled water. To generate a mucus layer, 1 mL of artificial mucus was added to each well above the gelatin layer. After that, 500 µL of GST, physical mixture, solid dispersion or optimum in situ gelling formulation groups (1 mg/mL) were added to the mucus layer and let to rest at room temperature. After 1 h of incubation at physiological temperature of 37 ± 0.05  C, the mucus layer was carefully removed, and the surface of the remaining gelatin layer was washed multiple times with 2 mL water. After heating the gelatin to 60 °C, it was mixed with acetonitrile and centrifuged at 6000 rpm for 20 min. Finally, the concentration of GST in the supernatant was determined by combining and vortexing methanol. After 100% solubilization, the GST concentrations in the clear solution were measured using a UV spectrophotometer at 260 nm [21, 22].

### Statistical analysis

One-way ANOVA followed by Tukey’s Multiple Comparison Test was used for the statistical analysis, and the data were expressed in terms of mean ± SD. In the statistical analysis, P < 0.05 was considered statistically significant, and the statistical analysis was done using Graph Pad Prism 4.0 software (Graph Pad Software San Diego, USA).

## Result and discussion

### Solid dispersion and GST content assay

The selected solid dispersion content assay was found to be 2.15 ± 0.01 mg per 5 mg of the solid dispersion for the GST. Overall, the GST content assay was 0.43 mg/mg of the dry powder.

### GST-GNS solid dispersion solubility assessment

The GST’s solubility in the GST-GNS solid dispersion was found to be more than 25 folds of the raw material in both media. In pH 1.2 and pH 6.8, the GST’s solubility in the physical mixture was more than 9 folds of the GST raw material. However, the GST’s solubility in the GST­GNS solid dispersion was higher than that of the GST one using physical combination, as indicated in Table 2. The enhanced solubility results of the GST-GNS solid dispersion compared to the free GST could be correlated to the hydrophilic interfaces of the hydrogen bonds and the GNS hydroxyl groups exposed due to the interactions between the GNS and GST as suggested in the FT-IR results. On the other hand, the dissolution enhancement using the solid dispersion was better than the physical mixture due to the higher interfacial tension that occurred using the physical mixture, in which, the hydrophilic interfaces on the physical mixture were not apparent as suggested by the FT-IR results. In general, the groups' solubilities at pH 6.8 were better than the 0.1N HCl, which could be due to the enhanced polarity of the GST hydroxyl groups in the simulated intestinal media [28, 29].

**Table 2:** GST, GNS, GST-GNS solid dispersion, and GST-GNS physical mixture solubilities in 0.1 N HCl and pH 6.8 media

Group	GST Solubility in 0.1 N HCl (mg/mL)*	GST solubility in pH 6.8 (mg/mL)^a^
GST	0.21 ± 0.01	0.30 ± 0.01
GST-GNS solid dispersion	5.79 ± 0.03	7.63 ± 0.14
GST-GNS physical mixture	2.07 ± 0.01	3.81 ± 0.04

Each value is the average of n = 3 ± SD

^a^For n = 3 ± standard deviation

### GST release analysis, gel characterization and storage stability testing

The GST-GNS solid dispersion was developed to explore the synergistic anticancer and penetration effects. When poor soluble GST is coated with GNS using the GST-GNS solid dispersion and kneading, it could enhance the dissolution and penetration of GST via ginsenosides present in GNS extract [13, 29]. Due to the extensive GST metabolism and poor intestinal absorption, an oral long-acting and gastric in situ gelling gel were optimized to encapsulate and localize the intestinal GST release of the GST-GNS solid dispersion, which the solid dispersion could show an enhanced dissolution and penetration compared to the raw GST particles [14]. Furthermore, the GNS coating of GST could lower the hepatic metabolism of GST after oral absorption due to the saturation of Cytochrome P450 by the GNS extract components [9, 15].

**Fig. 1:**
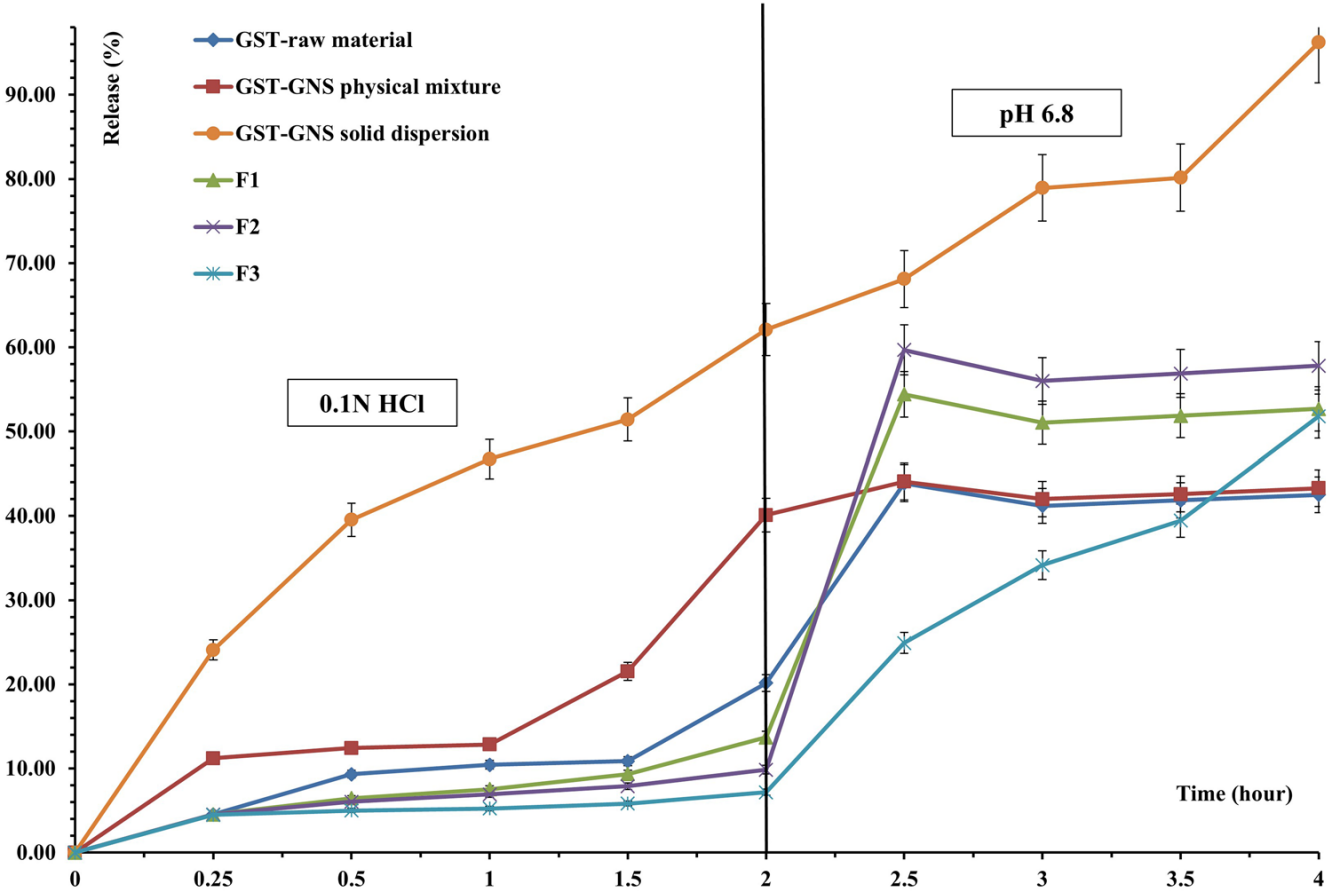
The GST release analysis for the GST-raw material, GST-GNS solid dispersion, GST-GNS physical mixture and different in situ gelling formulations (F1, F2 and F3)

The GST-GNS solid dispersion release rate of GST was faster than the free GST and GST-GNS physical mixture rates by more than two folds over the 4 h. In addition, the total solid dispersion GST release amounts were more than the free GST and physical mixture by more than 50.00 ± 0.03% over the dissolution time. Interestingly, the GST release difference between the solid dispersion and the other groups increased with time, especially at pH 6.8 (Fig. 1). As a result, the dissolution enhancement of GST using the solid dispersion was confirmed in the simulated gastric media and simulated intestinal media. Therefore, the solid dispersion dissolution enhancement might be due to the hydrogen bond interactions and the hydrophilic interfaces established in the solid dispersion, which was supported in the FT­IR [29]. Moreover, the PXRD and SEM results confirmed the amorphization and powder shape of the solid dispersion [16, 19, 20, 22].

For the comparison between the different insoluble gel-forming formulations containing the GST-GNS solid dispersion, the GST release analysis of the F1, F2 and F3 containing 2, 4, and 8 mg/mL, respectively, of CRG **(**Table 1**)** were studied using the Dissolution Rate (DR) for the gastric simulated media (Gas.DR) and the simulated intestinal media (Int.DR). The DR is the dissolved GST amount (mg) over 120 min of each phase using the following equation [6, 30].

$$\text{Dissolution \,rate }(\text{DR}) \left(\frac{mg}{min}\right)=\frac{GST \,average \,released \,amount \,after \,120 \,minutes}{120 \,minutes}$$

**Fig. 2:**
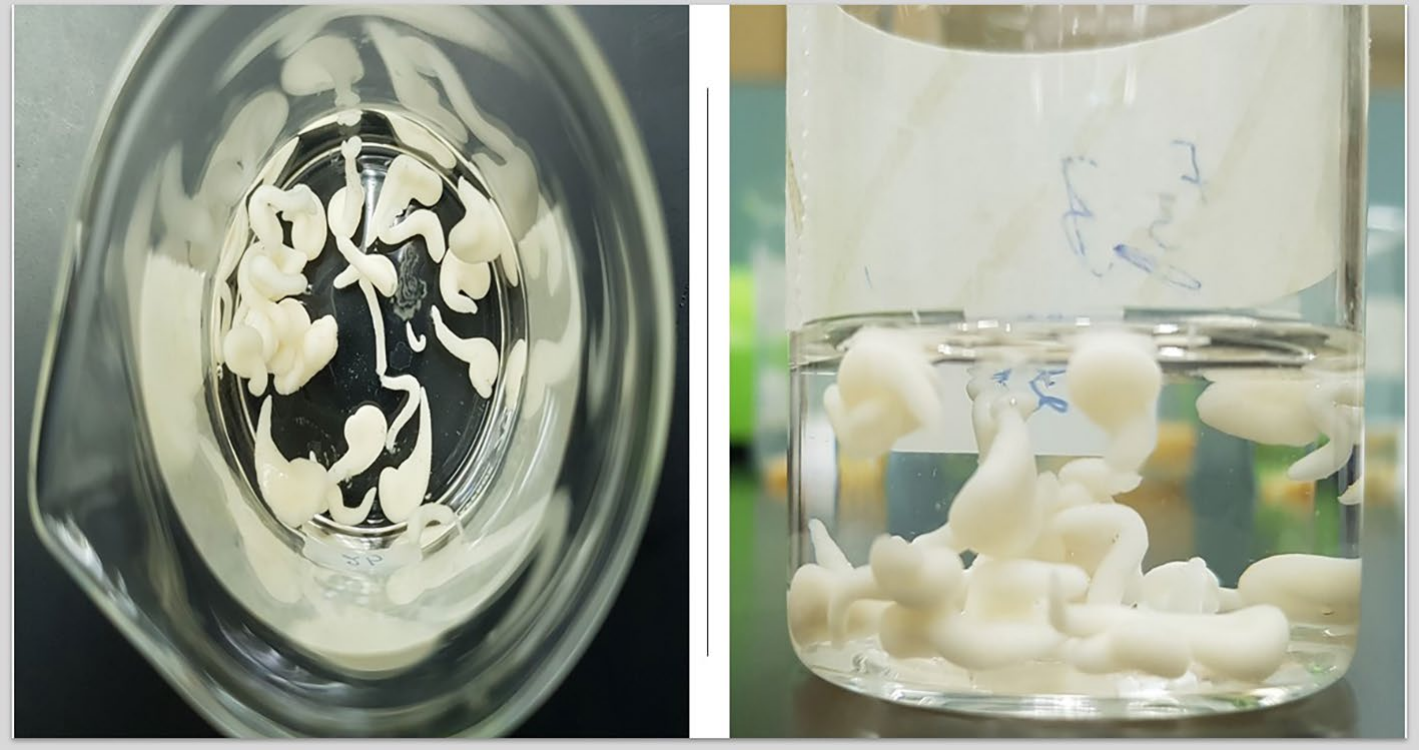
The most optimum gastric insoluble gel encapsulates the GST-GNS solid dispersion after 8 h of exposure to the 0.1N HCl

As shown in Fig. 1 and Table 3, the F3 release shows the lowest Gas.DR, and medial Int.DR values compared to the F1 and F2 formulations, which could contribute to the GST-GNS solid dispersion, CRG and NA hydrophilic interactions and hydrogen bond formation in the insoluble gel formation of the optimum NA, CRG, GNS and GST combinations in contact with 0.1N HCl that minimizes the GST release in the acidic media and controls the burst release in the intestinal media [23, 29]. The Gas.DR and Int.DR results of F3 formulation were reflected in the maximum release percentage values in the different media (Table 3). The F3 formulation showed the lowest value of the maximum GST release percentage values in the 0.1N HCl, and medial value for the pH 6.8 release compared to F1 and F2 formulations, which could support the intestinal localization of release. In coherence, the gel characterizations of F1, F2 and F3 are listed in Table 4**.** The gel characterizations of the F3 formulation showed the most resilient, strongest, and highest density compared to the F1 and F2 formulations. The F3 as the most optimum formulation’s gel after 8 h of exposure to the 0.1N HCl was illustrated in Fig. 2 [23].

**Table 3:** GST release parameters in 0.1N HCl and media of pH 6.8 from different gastric insoluble gels forming formulations of GST-GNS solid dispersion

Formulation	Gas.DR (mg/min)	Int.DR (mg/min)	Maximum 0.1 N HCl release (%)	Maximum pH 6.8 release (%)
F1	68.52 ± 0.02	194.92 ± 0.02	13.70 ± 0.67	38.99 ± 0.07
F2	49.62 ± 0.04	239.66 ± 0.04	9.85 ± 0.15	47.93 ± 0.09
F3	35.81 ± 0.05	223.23 ± 0.06	7.16 ± 0.10	44.65 ± 0.06

**Table 4:** Gastric insoluble gels characterizations

#	Gel formation rate	Gel position	Gel weight (g) ± SD^a^	Gel volume (ml) ± SD^a^	Gel strength (g) ± SD^a^	Gel resilience (min)
						Median
F1	After 15 s	Floated	0.71 ± 0.22	5.71 ± 0.42	8.31 ± 0.33	20
F2	After 5 s	Floated and sedimented	0.93 ± 0.18	7.20 ± 0.16	11.24 ± 0.19	95
F3	After 2 s	Floated and sedimented	1.33 ± 0.41	8.21 ± 0.17	18.11 ± 0.14	≥ 120

^*^For n = 3 ± SD

Regarding the storage stability testing of the most optimum in situ gelling formulation, the release of the F3 at zero-time, 2 weeks and 4 weeks storage time were demonstrated in Table 5 and Supplementary Figure. In general, the different profiles show the same pattern of release. However, the zero-time’s Gas.DR, Int.DR, maximum gastric and intestinal release values were lower than the 2 week’s values by 8 mg/min, 14 mg/min, 1.5% and 3%, respectively. In the same manner, the zero-time’s Gas.DR, Int.DR, maximum gastric and intestinal release values were lower than the 4 weeks values by 11 mg/min, 19 mg/min, 2.5% and 4%, respectively. The storage and ageing stability testing show an increase in the release rates, especially after 4 weeks of storage of at least 20% and 5% increase of the gastric and intestinal dissolution rate, respectively. This could be due to the time effect on the NA and CRG swelling, hydrodynamic stability and gelling ability to encapsulate the GST molecules, especially in the simulated gastric media [23, 36, 37]. The storage release stability testing could suggest shaking before use and, most importantly, the immediate reconstitution of the GST-GNS solid dispersion with CRG gel before administration and up to week use.

**Table 5:** Storage stability testing parameters in 0.1 N HCl and media of pH 6.8 from the optimum F3 insoluble gels forming formulation of GST-GNS solid dispersion at Zero-time, 2 weeks, and 4 weeks storage

Storage time	Gas.DR (mg/min)	Int.DR (mg/min)	Maximum 0.1 N HCl release (%)	Maximum pH 6.8 release (%)
Zero time	35.81 ± 0.05	223.23 ± 0.06	7.16 ± 0.10	44.65 ± 0.06
2 weeks	43.15 ± 0.13	237.68 ± 0.21	8.63 ± 0.10	47.54 ± 0.08
4 weeks	46.75 ± 0.33	242.35 ± 0.24	9.35 ± 0.05	48.47 ± 0.37

Each value is the average of n = 3 ± SD

### GST-GNS solid dispersion characterizations

#### FT-IR analysis of GST-GNS

**Fig. 3:**
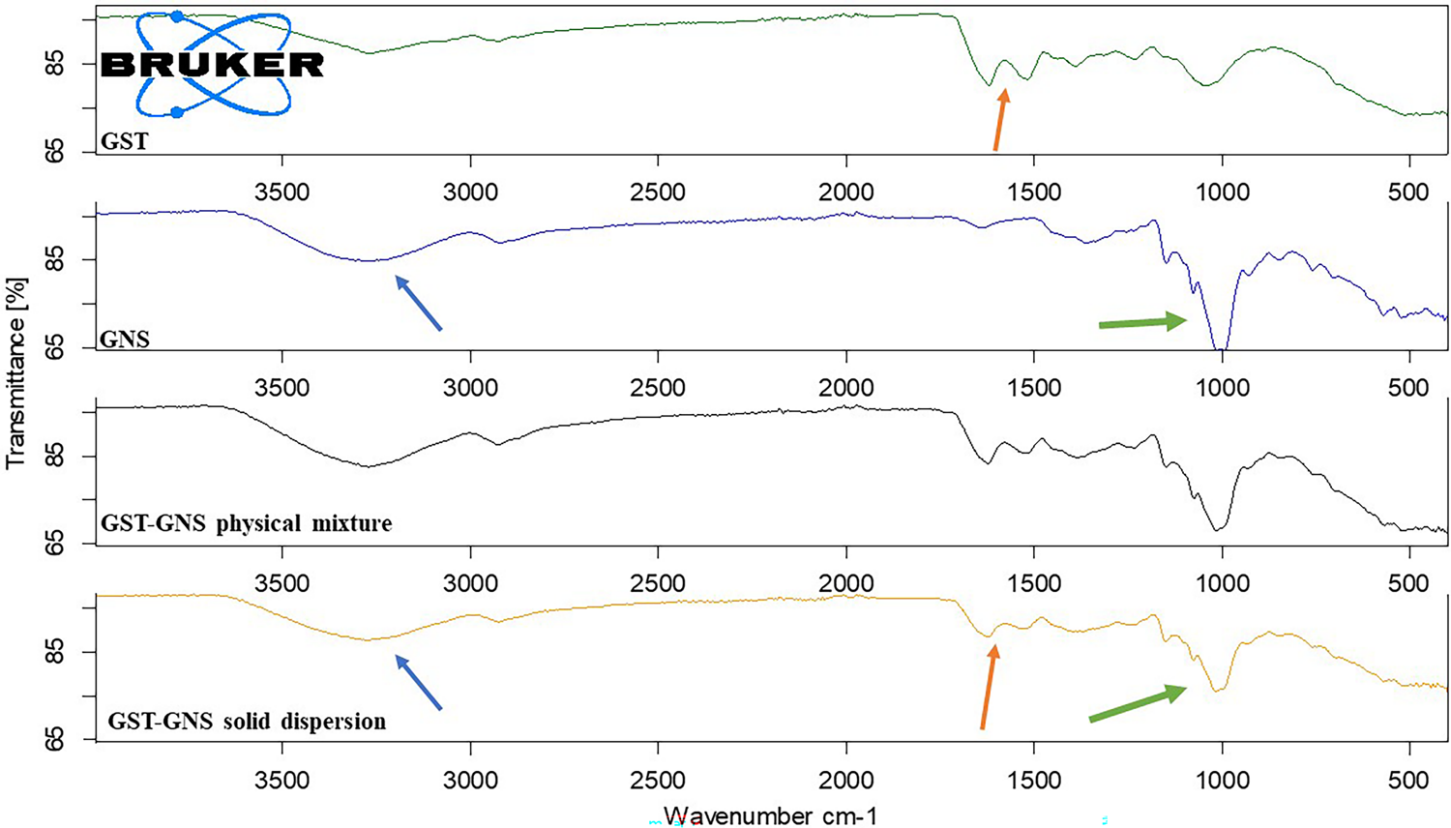
The FT-IR spectra for the genistein (GST), ginseng (GNS), GST-GNS physical mixture and GST-GNS solid dispersion

As shown in Fig. 3, the GST and GNS spectra comply with the literature [19, 20, 31]. Moreover, the physical mixture shows the GNS peaks and GST peaks with lower intensity due to the dilution effect [32]. On the other hand, the GST-GNS solid dispersion spectrum shows the GNS peaks more than the GST peaks, which might be related to the GNS surface coating of the GST core as the related peaks were marked in Fig. 3 with the same colour [29, 33]. The carbonyl (C = O) at 1600 cm^−1^ and aromatic (C = C) stretching of GST at 1500 cm^−1^ were diminished in the solid dispersion spectrum to confirm the GST inner molecules' orientation and dilution with GNS coating. The FT-IR analysis confirms the GNS’ ginsenosides coating of the GST [33].

#### Crystallinity analysis using PXRD

**Fig. 4:**
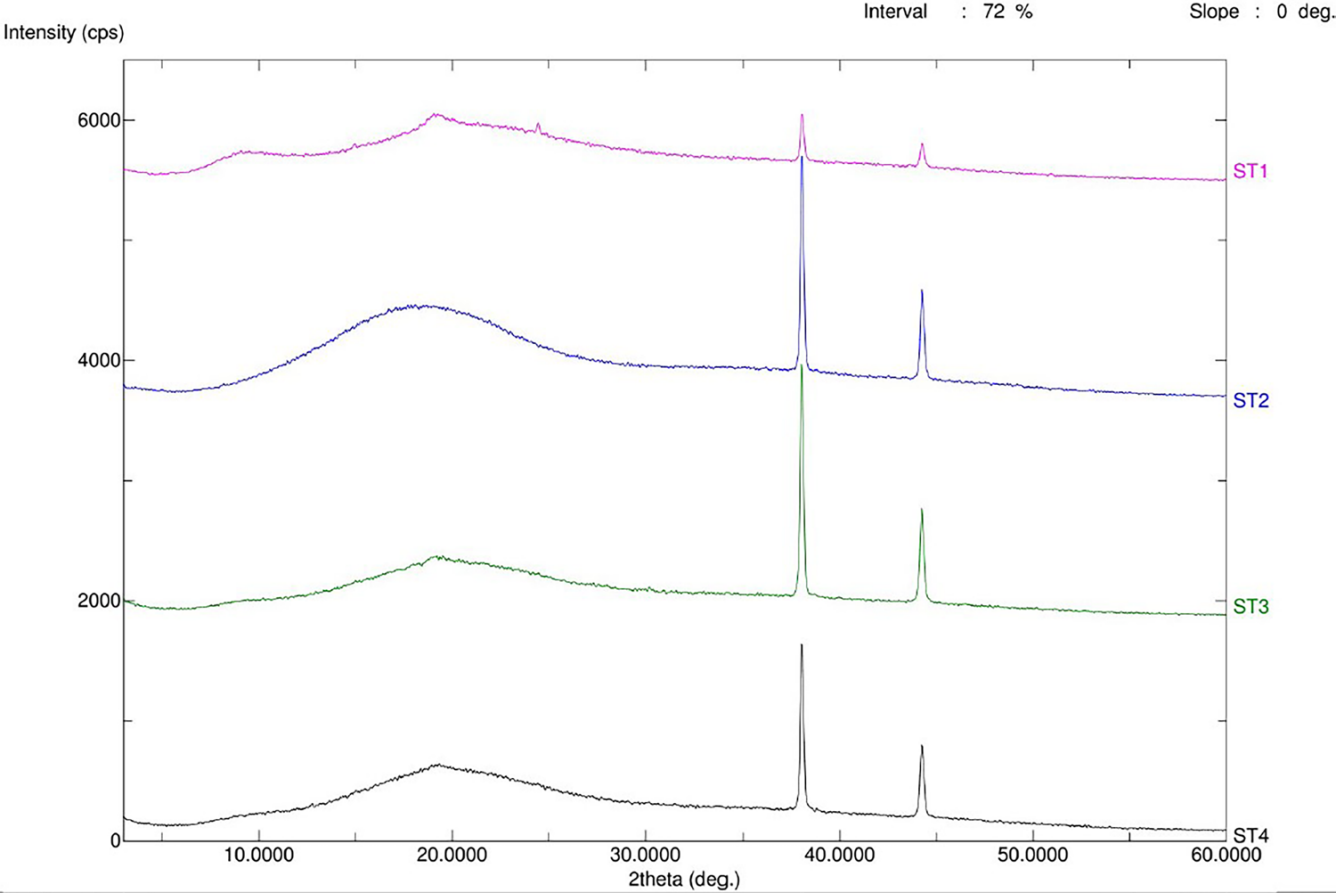
The PXRD diffractograms of the genistein (GST, ST1), ginseng (GNS, ST2), GST-GNS physical mixture (ST3) and GST-GNS solid dispersion (ST4)

As shown in Fig. 4, the GNS (ST-2) diffractogram complies with the noisy amorphous diffractogram with characteristic peaks at 2 Theta values of 37.48°, 44.54° and broad peak from 9.70° to 25.78°. Moreover, the GST (ST-1) diffractogram bears the amorphous character with characteristic peaks at 2 Theta values of 37.48°, 44.54° and broad peak from 7.02° to 9.70°. On the other hand, the physical mixture (ST-3) and solid dispersion (ST-4) diffractograms show noisy amorphous peaks with two characteristic peaks at 2 Theta values of 37.84° and 44.54°, which was similar to the GST and GNS diffractograms. Finally, the solid dispersion diffractogram shows a similar diffractogram of the GNS with a broad 2 Theta peak from 13.72° to 24.44°. In addition, the solid dispersion diffractogram shows a similar amorphous character to the GNS diffractogram due to the dominant GNS coating [29, 34].

#### Morphology and dispersion investigations

**Fig. 5:**
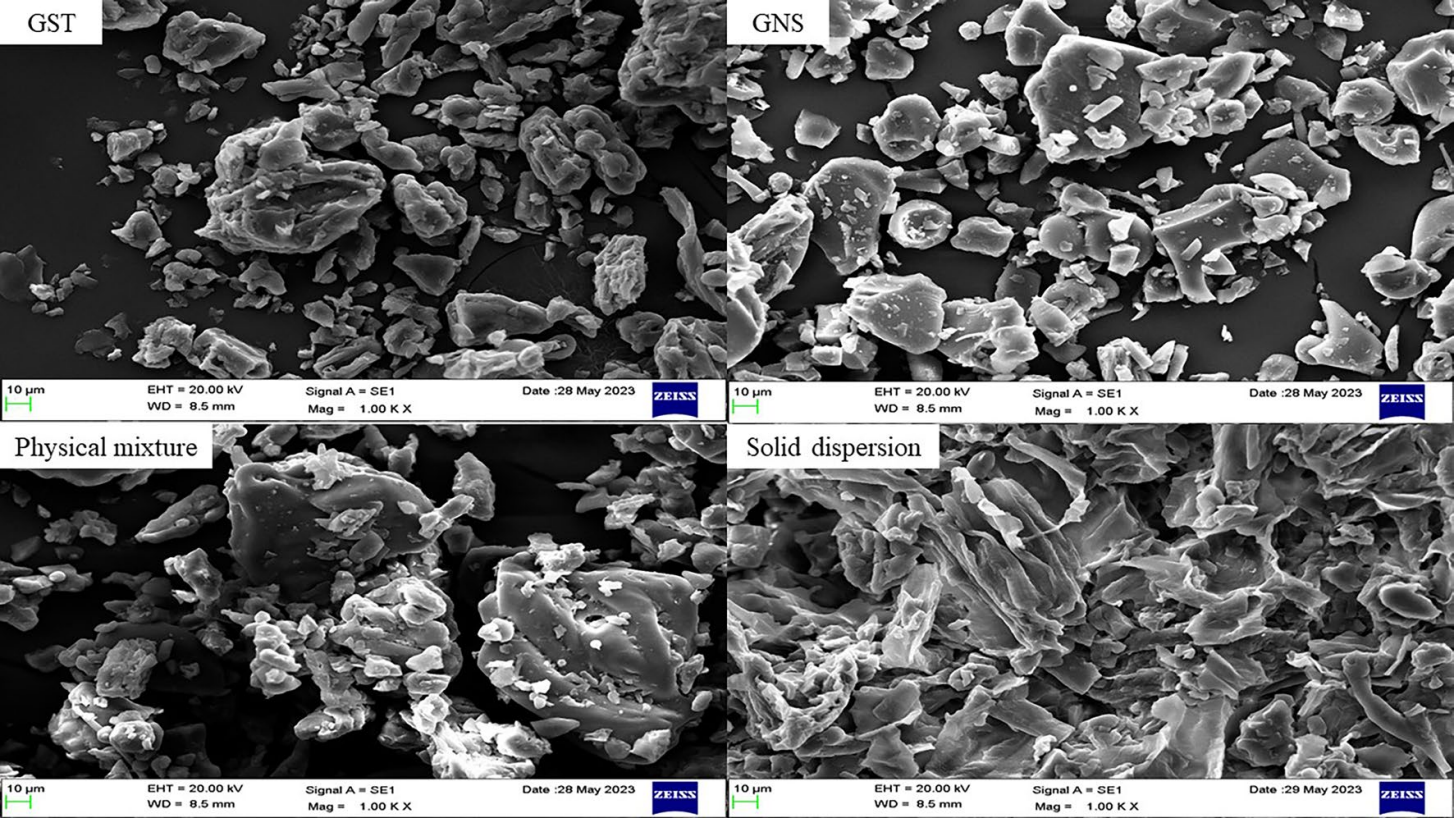
The SEM images of the genistein (GST), ginseng (GNS), their physical mixture and solid dispersion at a magnification of 1000 X and voltage of 20.00 kV

As shown in Fig. 5, the GST has a more irregular powder bed and wider particle size distribution than the GNS at a magnification power of 1000 X and scale bar of 10µm. Moreover, the physical mixture of GST-GNS combines the raw material powder shapes at the same former magnification power and scale. On the other hand, the GST-GNS solid dispersion gives different powder characteristics than the GST, GNS, or the physical mixture. The solid dispersion powder’s structure and distribution are more uniform and show smooth shapes and narrow particle size distribution than the GST and physical mixture [29].

### Assay for the cell viability and penetration study

**Fig. 6:**
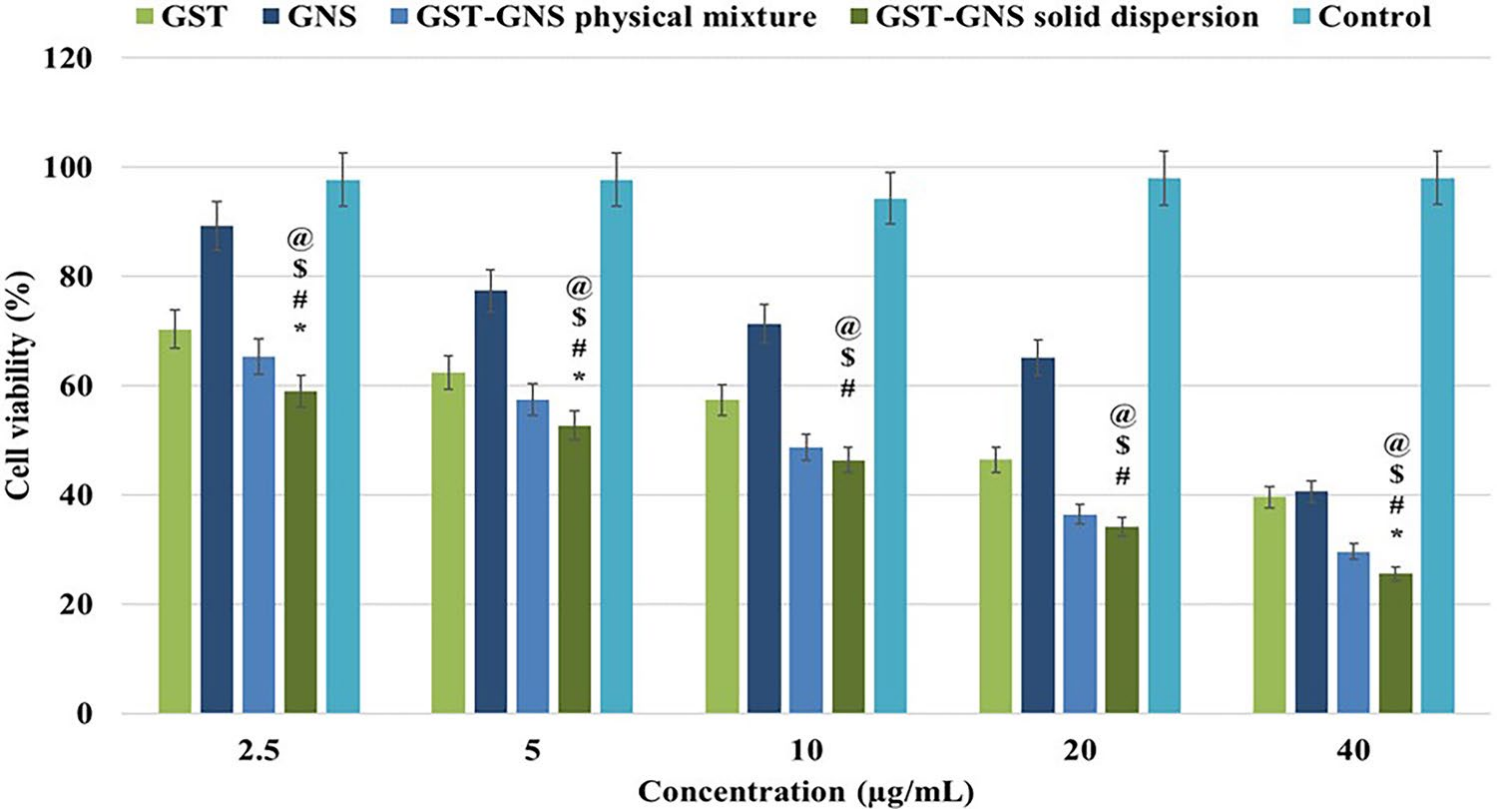
The MTT assay of the GNS, GST, their physical mixture, and solid dispersion groups. One-way ANOVA was to determine the significance between the GST-GNS solid dispersion with GST (@), GNS ($), physical mixture (*), and control (#), nwhich the P-value of less than 0.05 was considered significant

**Fig. 7:**
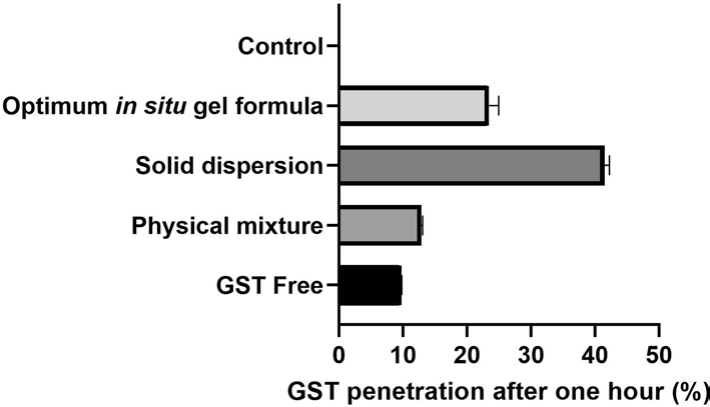
The GST penetration percentage of the GST-free, GST-GNS physical mixture, GST-GNS solid dispersion and optimum in situ gelling formulation groups

Cell viability or MTT assay is one of the decisive parameters for estimating the anticancer potential of the drug on the cellular level. In the MTT assay, upon exposure to MTT dye and various drugs, viable cells get coloured due to DNA fragmentation, and the absorbance is measured [35]. Quantifying absorbance and subsequent calculation gives a solid background about the viability and provides the suitable concentration (50%-Inhibition Concentration”IC_50_″) that exhibits an anticancer effect [35]. In the present study, when MCF-7 BC cells were treated with different formulations, it was found that IC_50_ values of GNS and GST were 33.38 ± 1.57 and 22.33 ± 0.74 µg/mL, respectively. The IC_50_ values of the physical mixture and GST-GNS solid dispersion were 12.67 ± 1.57 and 7.90 ± 0.13 µg/mL, respectively **(**Fig. 6**)**. Based on the MTT assay, it could be inferred that the GST-GNS solid dispersion is almost two times more potent than the physical mixture, three times more powerful than GST, and four times more potent than GNS. Additionally, anticancer potency of GST was found to be more than the GNS, and less than the physical mixture. The superior cytotoxicity of the GST-GNS solid dispersion could be due to their synergistic effects, GST dissolution and penetration enhancements [36]. As in Fig. 7, the percentage of GST penetration after 1 h of exposure for the solid dispersion was 4.0 and 3.3 folds more than the GST-free and physical mixture penetration values, respectively. This could be attributed to the high membrane affinity and miscibility of the surface functionalities exposed in the GST-GNS solid dispersion, which could be due to the solid dispersion and hydrophilic interface arrangements [29]. On the other hand, the physical mixture enhanced penetration compared to the raw GST group could be due to the GNS’s associated glycosides of saponins-like effects, which might disturb the membrane integrity. However, the optimum in situ gel penetration of GST was lower than the GST-GNS solid dispersion due to the long-acting gelling system that the GST encapsulated in [37].

## Conclusions

This study developed a solid dispersion between the GST and GNS, which showed distinctive interactions, morphology, and amorphicity. As a result, these unique properties were the foundation of the enhanced solid dispersion dissolution in the simulated gastrointestinal tract media over time. Furthermore, the enhanced dissolution, antitumor synergism, and enhanced penetration between the GST and GNS enhance the GST efficacy after oral administration to reach the breast tissue as a high perfusion tissue for GST. In addition, the development of the optimized gastric in situ gelling system loaded with the GST-GNS solid dispersion showed release preference and controlled GST release behaviour toward the intestinal media. Thus, the novel oral genistein long-acting gel encapsulating the GST-GNS solid dispersion would be suggested for an improved therapeutic approach for BC or colorectal cancer using the ameliorated bioavailability and synergized anticancer efficacy.

### Supplementary Information

Additional file1 (JPG 327 KB)

## Data Availability

The data and materials are available. It can be given on request.
