# Supplementary figures and images for: Preparation, optimization, and characterization of genistein-ginseng long-acting polymeric gel as a breast cancer treatment alternative
Source: Discov Oncol. 2024 Jul 3;15:257. doi: 10.1007/s12672-024-01132-8 (PMC11222347; doi:10.1007/s12672-024-01132-8)

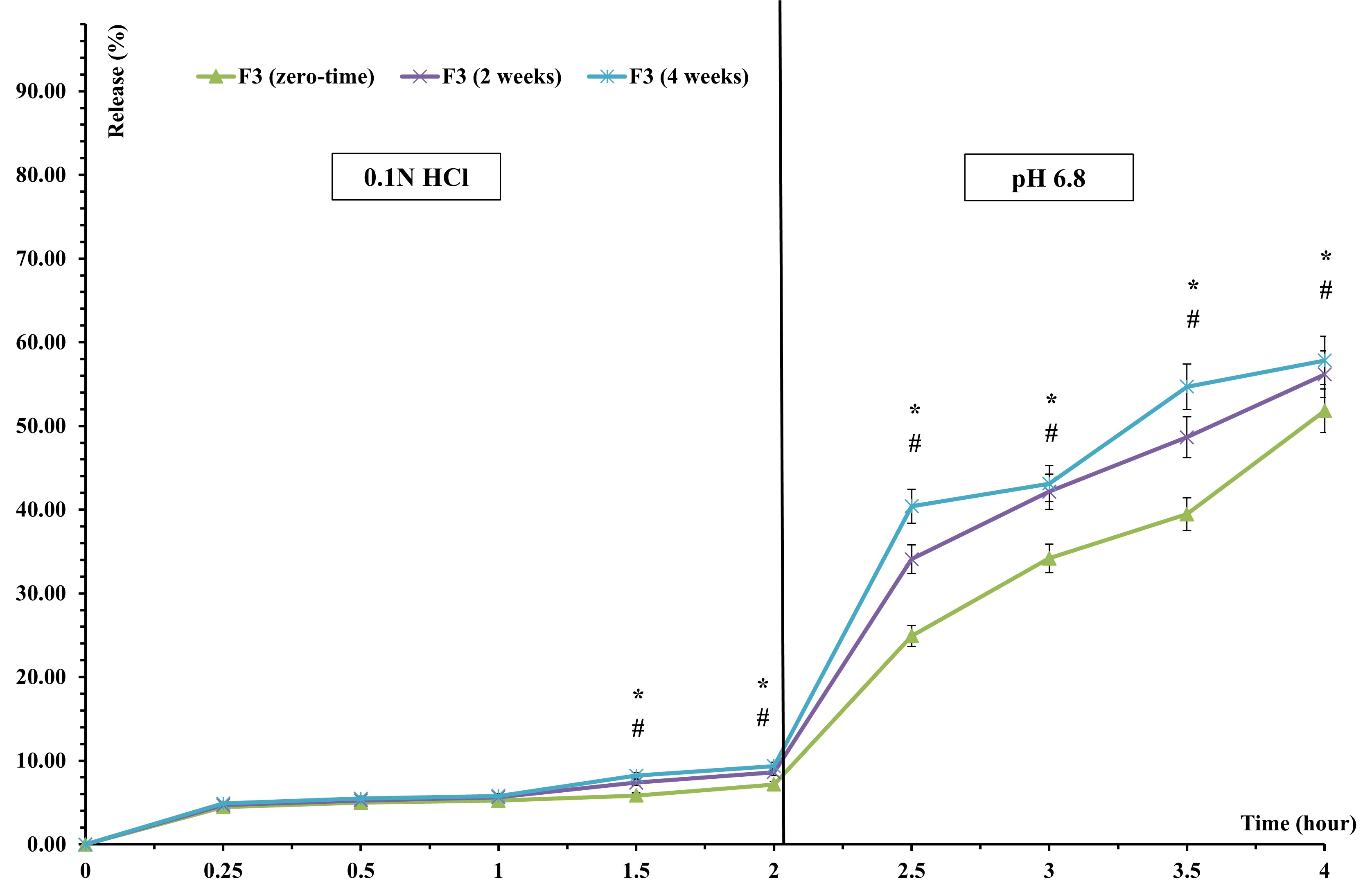

Supplement: Supplementary file 1 — Additional file1 (JPG 327 KB) [file 12672_2024_1132_MOESM1_ESM.jpg]
